# Case Definitions for Conditions Identified by Newborn Screening Public Health Surveillance

**DOI:** 10.3390/ijns4020016

**Published:** 2018-05-09

**Authors:** Marci K. Sontag, Deboshree Sarkar, Anne M. Comeau, Kathryn Hassell, Lorenzo D. Botto, Richard Parad, Susan R. Rose, Kupper A. Wintergerst, Kim Smith-Whitley, Sikha Singh, Careema Yusuf, Jelili Ojodu, Sara Copeland, Cynthia F. Hinton

**Affiliations:** 1 Department of Epidemiology, Colorado School of Public Health, University of Colorado Anschutz Medical Campus, Aurora, CO 80045, USA; 2 NewSTEPs, Newborn Screening Technical assistance and Evaluation Program, A Program of the Association of Public Health Laboratories, Silver Spring, MD 20910, USA; 3 Health Resources and Services Administration, Maternal Child Health Bureau, Division of Services for Children with Special Health Needs, Genetic Services Branch, Rockville, MD 20852, USA; 4 New England Newborn Screening Program, University of Massachusetts Medical School, Worcester, MA 01605, USA; 5 Division of Hematology, University of Colorado School of Medicine, University of Colorado Denver Anschutz Medical Campus, Aurora, CO 80045, USA; 6 Department of Pediatrics, University of Utah School of Medicine, Salt Lake City, UT 84132, USA; 7 Department of Pediatrics, Harvard Medical School Department of Pediatric and Newborn Medicine, Brigham and Women’s Hospital, Boston, MA 02115, USA; 8 Endocrinology and Pediatrics, Cincinnati Children’s Hospital Medical Center, University of Cincinnati College of Medicine, Cincinnati, OH 45229, USA; 9 Division of Endocrinology, Department of Pediatrics, School of Medicine, University of Louisville, Louisville, KY 40202, USA; 10 Division of Hematology, Children’s Hospital of Philadelphia, Department of Pediatrics, Perelman School of Medicine, Philadelphia, PA 19104, USA; 11 Association of Public Health Laboratories, Silver Spring, MD 20910, USA; 12 Palo Alto Medical Foundation, Daly City, CA 94015, USA; 13 National Center on Birth Defects and Developmental Disabilities, Centers for Disease Control and Prevention, Atlanta, GA 30341, USA

**Keywords:** newborn screening, case definitions, short-term follow up, public health surveillance

## Abstract

Newborn screening (NBS) identifies infants with rare conditions to prevent death or the onset of irreversible morbidities. Conditions on the Health and Human Services Secretary’s Recommended Uniform Screening Panel have been adopted by most state NBS programs, providing a consistent approach for identification of affected newborns across the United States. Screen-positive newborns are identified and referred for confirmatory diagnosis and follow-up. The designation of a clinically significant phenotype precursor to a clinical diagnosis may vary between clinical specialists, resulting in diagnostic variation. Determination of disease burden and birth prevalence of the screened conditions by public health tracking is made challenging by these variations. This report describes the development of a core group of new case definitions, along with implications, plans for their use, and links to the definitions that were developed by panels of clinical experts. These definitions have been developed through an iterative process and are piloted in NBS programs. Consensus public health surveillance case definitions for newborn screened disorders will allow for consistent categorization and tracking of short- and long-term follow-up of identified newborns at the local, regional, and national levels.

## 1. Introduction

Newborn screening (NBS) is acknowledged to be one of the top 10 US public health achievements in the first decade of the new millennium [1]. NBS conditions may not be clinically apparent at birth, but without immediate intervention, they may lead to developmental disabilities, severe illness, or premature death. NBS encompasses both laboratory testing for various disorders using dried bloodspots (DBS) collected on filter paper cards and point-of-care testing for hearing loss and critical congenital heart defects. Approximately 4 million newborns are screened annually in the United States, with disability or death averted for thousands of newborns due to early detection through NBS [2].

### 1.1. History of Public Health Newborn Screening in the United States

NBS for phenylketonuria (PKU) was introduced in 1962 [3], using filter paper to collect and transport DBS from newborns [4]. State public health systems adopted NBS for PKU nationally. Over time, additional disorders added for screening through state-based processes included aminoacidopathies and galactosemia in the 1960s [5], congenital hypothyroidism in the 1970s [6], and biotinidase deficiency and congenital adrenal hyperplasia in the early 1980s. Screening for hemoglobinopathies was initiated in some states in the 1970s, with subsequent adoption in all states through the 1990s and 2000s [7]. Early investigations into the feasibility of screening for cystic fibrosis (1982) [8] preceded implementation of statewide screening with some states screening in the 1980s–1990s and the remaining states implementing screening for this disorder in the 2000s. The introduction of tandem mass spectrometry technology made it possible to test for numerous conditions in parallel, which was a major factor in the rapid expansion of NBS panels during the 2000s [9]. In an effort to create uniformity in NBS across the United States, the Recommended Uniform Screening Panel (RUSP) for NBS conditions was published in 2006 with 29 core conditions and 25 secondary conditions [10] and was officially adopted by the US Secretary of Health and Human Services in 2010. The RUSP has expanded to 34 conditions as of February 2016. Two of the conditions on the RUSP, critical congenital heart defects and hearing loss, are screened in the nursery and referred to as “point-of-care” NBS, while the remaining conditions are screened using DBS at a central laboratory within a state or region [11]. Currently, all states require screening for at least 26 of the 34 recommended conditions, and standards for laboratory practices have been developed [12].

### 1.2. Rationale for Surveillance Case Definitions

The case definitions presented here are new and not the update of any previously published standard case definitions. The absence of case definitions has been identified as a barrier to combining data across state and regions to conduct meaningful analysis on rare conditions [13]. Historically, each state has relied on case determination made by clinical consultants whose principal responsibility is the evaluation and treatment of patients at risk. Thus, local availability of diagnostic tests, extent of subspecialty expertise, individual clinical judgment, and variability in monitoring outcomes all contribute to variability in case definition. Implementation of case definitions from the public health perspective would not change clinical diagnosis or care but would provide a common nomenclature across programs for evaluation of the frequency of case identification, including the timing of diagnosis and care. The lack of standardized measures for diagnosis makes it difficult to determine an accurate frequency of newborns with conditions on the RUSP identified by NBS and renders comparison of detection rates inaccurate [14]. More importantly, the use of standardized surveillance public health case definitions would allow for meaningful tracking of cohorts of individuals with conditions over time to better understand outcomes following an NBS diagnosis [15].

### 1.3. Rationale for Case Definitions for Each Condition Group

Each group of conditions is presented below with an example illustrating the complexities of clinical diagnosis, demonstrating the need for public health surveillance case definitions. The multiple etiologies and varying phenotypes of each of these conditions can make it challenging for public health programs to classify diagnosed cases uniformly. While clinical diagnoses are made based upon an individual practitioner’s assessment of the infant’s symptoms and diagnostic testing, public health programs need to characterize infants based on standard and consistent criteria. Public health surveillance activities are developed toward achieving health equity and the results of the activities can inform resource allocation, highlighting the need for consistent case definitions in NBS.

#### 1.3.1. Metabolic Conditions

NBS for identification of metabolic disorders is performed by identifying values for biochemical markers that fall outside of the normal physiological range. These biochemical marker values may indicate a possible enzyme defect in one of the metabolic pathways involved in normal cellular function. However, some markers are nonspecific. Metabolites may be elevated due to dietary factors, from immaturity of the metabolic pathway or as a result of decreased amounts of normally functioning enzymes. Therapies can vary markedly depending on specific phenotypic variants. Thus, it is crucial to confirm the presence of a true metabolic disorder in order to best counsel the family, manage dietary or lifestyle adjustments, and determine the need for medical intervention. This may require multiple types of testing, including determining analyte concentrations, enzyme function, and DNA sequence variants. The level of diagnostic testing will determine the level of confidence in diagnosis.

#### 1.3.2. Hemoglobinopathies

In the absence of DNA analysis on the infant, most hemoglobinopathies are clinically defined and require the interpretation of results from hemoglobin electrophoresis and Complete Blood Count (CBC) findings (e.g., the presence or absence of microcytosis), sometimes in the setting of carrier testing of the biologic mother and father. Many conditions do not fully manifest until abnormal hemoglobins are generated during the transition from the production of fetal to adult hemoglobin. However, fatal infectious complications of sickle cell disease related to loss of splenic function may occur in early infancy, which can be prevented with prophylactic penicillin beginning by two months of age. While variant hemoglobins can be detected at birth, the diagnosis of some hemoglobinopathies may be difficult before one year of life, when the conversion from fetal to variant hemoglobin has been made, with a corresponding change in the CBC and hemoglobin electrophoresis results. For example, it would be very difficult to distinguish S-hereditary persistence of fetal hemoglobin, which is a very mild sickle cell syndrome, from Hemoglobin SS, a potentially more severe state in the first year of life. Appropriately rigorous DNA testing is often necessary to establish and characterize the thalassemias.

#### 1.3.3. Cystic Fibrosis (CF)

The diagnostic gold standard for cystic fibrosis is the sweat test. However, given both the difficulty in obtaining adequate quantities of sweat and the variability in sweat chloride concentration in infants, it can be difficult to confirm a diagnosis even in those with CF. When the sweat chloride is <60 mmol/L, the diagnosis is inconclusive and requires additional testing, such as mutation analysis of the cystic fibrosis transmembrane conductance regulator (CFTR) gene, fecal elastase determination, and clinical monitoring over time. In many states, a genotype with two mutations may be reported as part of the NBS, but in the absence of a confirmatory sweat test or repeat genotyping on the infant, a diagnosis cannot be confirmed. Infants with only one mutation detected require sweat chloride testing to distinguish between carrier status and affected status. Finally, clinical consequences of each of the over 2000 known CFTR mutations are known to vary, and there may be varying interpretations regarding the impact of those mutations by clinicians [16]. Hypertrypsinogenemic infants identified as having CFTR mutations of varying clinical consequence in conjunction with inconclusive sweat chloride concentration may be assigned a diagnostic place holder known in the United States as CFTR Related Metabolic Syndrome (CRMS) and in Europe as Cystic Fibrosis Screen Positive, Inconclusive Diagnosis (CFSPID).

#### 1.3.4. Endocrinology

Both congenital hypothyroidism (CH) and congenital adrenal hyperplasia (CAH) have multiple etiologies and may manifest phenotypes of varying levels of severity. When a CH diagnosis is missed and goes untreated, it can result in developmental delay and disability. Undetected salt-wasting CAH may lead to dehydration and death. While it is known that prompt initiation of thyroid hormone treatment to restore normal thyroid stimulating hormone (TSH) and free thyroxine (FT4) concentrations by one month of age can permit normal intellectual development and linear growth [17,18], the diagnosis of primary CH can be complicated by the multiple stages of diagnostic testing and evaluation. Primary CH must be differentiated from central (secondary/tertiary) CH through additional laboratory and clinical observations. Central CH will not be detected on a primary TSH screen because the TSH level is not typically elevated but may be detected on a primary thyroxine (T4) screen. Confirmation relies on clinical identification of other associated features, such as midline facial/ brain abnormalities, hypoglycemia, microphallus, or other hormone abnormalities.

Salt-wasting CAH, caused by 21-hydroxylase deficiency, must be differentiated from simple virilizing (which typically does not require intervention in the newborn period) or late onset CAH (which typically does not require intervention in the newborn period). The distinction can be made by evaluating the extent of elevation of the hormone marker 17-hydroxy-progesterone through a sensitive diagnostic laboratory test. Concurrently, the pediatrician (alerted by the abnormal screening test) establishes close monitoring of the infant’s clinical and electrolyte status until definitive results become available.

#### 1.3.5. Immunology

Severe combined immunodeficiency (SCID) comprises at least 13 independent genetic conditions, all of which present with low or absent functional T cells. The newborn screen for SCID uses a marker of T cell functionality, and its absence indicates risk for SCID. Other non-SCID disorders may also show absence of this marker, and other non-SCID conditions on the differential diagnosis will be identified in the course of further evaluation. The overlapping features among SCID, leaky SCID, Omenn syndrome, and non-SCID disorders can sometimes be distinguished either with the help of genotyping, clinical monitoring, or both, in order to determine if urgent treatment is needed. The variability in access to expertise and resources yields a compelling argument for ascertaining the certainty of the diagnosis (SCID, leaky SCID, Omenn syndrome, and non-SCID T-cell lymphopenia).

## 2. Materials and Methods

This is the first time NBS case definitions for public health surveillance have been created. There are no prior models to determine the certainty of cases within the context of a public health screening program in a uniform way. Robust surveillance is critical as many NBS conditions require lifelong care. Case definitions are an important foundation for the continuous process of tracking these conditions, assessing outcomes, and improving treatments, with the ultimate goal of contributing to optimal growth, development, and lifelong health of newborns with such rare conditions.

### 2.1. Expert Workgroups

Clinical experts were recommended by the Health Resources and Services Administration (HRSA) funded grantees, other federal agencies working on NBS projects, including the Centers for Disease Control and Prevention (CDC) and the National Institutes of Health (NIH), and by relevant professional organizations (Expert participants and their organizational affiliations are listed in the Supplementary Materials of this report) The expert workgroups were Metabolic, Endocrinology, Hematology, Immunology, and Pulmonology. Workgroup members had expertise in NBS in the following areas: clinical, laboratory, or epidemiology and public health. Workgroup clinical members were all board certified in their area of expertise, with clinical experience and practice in their field. Due to limited resources, the workgroups were US-based, but the tools were developed to be amenable for implementation in other countries. Although a number of federal partners were involved, a federal advisory committee did not oversee the development of these recommendations.

### 2.2. Data

#### 2.2.1. Literature Search

Biomedical literature was searched by HRSA staff for evidence of previous surveillance case definitions using the MEDLINE database of the National Library of Medicine. The search dates were from January 1966 through May 2011. The criteria to identify studies from the literature that had utilized previous NBS case definitions were

- Medical subject headings of “newborn screening” “case definitions” and “surveillance”;
- English language;
- Peer reviewed with original data (not review articles).

Following this search criteria, there were no references identified that defined or used standard case definitions for NBS conditions. The literature review produced no relevant studies to NBS conditions. Therefore, we expanded the literature search in a less systematic way to identify examples of case definitions in other fields.

#### 2.2.2. Information from State and Regional Programs

In addition to the search for surveillance case definitions, all state NBS programs were queried to determine if individual programs had developed models for NBS case definitions. The catalogued resources of the National Coordinating Center (NCC) for Regional Genetic Service Collaboratives (RCs) were searched for regional definitions or practice guidelines [19]. Although no standard case definitions were found, several state programs and Regional Genetic Service Collaboratives’ activities provided the basis for developing the minimum necessary testing elements required in order to establish a diagnosis and subsequent standard case definition. These sources were the Mountain States Regional Genetics Collaborative Disease Specific Care Plans [20], the Region 4 Stork (R4S) Data System [21], the California Metabolic Group case definitions [22], the New York–Mid-Atlantic Consortium Collaborative (NYMAC) clinical guidelines [23], and the American College of Medical Genetics and Genomics (ACMG) ACT Sheets consensus-based guidelines [24]. These resources were not designed to provide standardized public health surveillance case definitions: The R4S program informs screening parameters to improve detection of metabolic conditions. Other regional guidelines are intended to guide primary care providers in the diagnostic workup of newborns with abnormal NBS results, and they provide limited utility in developing surveillance case definitions. In contrast, the case definitions developed through the process we describe here were designed to enable public health programs to systematically collect standardized data.

## 3. Results

### 3.1. Case Definition Model

HRSA staff identified examples of case definition models for other disorders or body systems to inform the categorical designation for the diagnosis of NBS disorders [22,23,25,26,27,28,29,30]. However, a systematic review of the literature for general case definition models was not performed. The models selected for discussion are described below.

The expert workgroups received a copy of the literature that informed the case definition models. The workgroup members used the regional and state materials described above, their own actual NBS case data, and the following case definition models before the in-person meeting. Pre-meeting discussions were held in a secure online location. The three model types considered were:

1. Tiered model: Tier one would consist of cases that no expert would dispute as confirmed disease. Subsequent tiers would focus on ambiguous cases that may or may not be considered true disease, based on the extent of the diagnostic workup and accompanying results [25,26,27].
2. Quantitative model: Points would be assigned based on diagnostic test criteria that would be dependent upon the performance of particular laboratory tests, and the interpretation of those results based upon a predetermined scale [28,29].
3. Diagnostic models: These models were based on the previously published regional or state NBS projects that were developed to assist clinicians in caring for newborns identified with conditions through NBS (CDC 4-States Pilot Project [30], NYMAC Diagnostic Guidelines [23], and California Metabolic Group case definitions [22]).

Experts identified the components of the diagnostic workup that may be commonly used in order to confirm or rule out a specific diagnosis following an abnormal newborn screen. De-identified cases of specific diagnoses and of differing severity were discussed and the models were applied. Experts were asked to discuss the strengths and weaknesses of the respective models and propose possible solutions.

Overview of expert deliberation process: Expert groups met through conference calls and shared web-based workspaces. Conference calls and web-based interactions were conducted to establish goals prior to an in-person meeting in Washington, DC in June 2011. The metabolic workgroup met in person again in February 2012. All expert workgroups met independently, with a federal staff member as a facilitator, to discuss each proposed case definition model. Each expert workgroup was given the discretion to investigate the models that they felt would best fit their disorder(s). Definitions were decided by consensus following active debate and discussion. Initial work was completed within a 13 months period beginning with the in-person meeting and followed by monthly webinars.

The quantitative model was initially considered by the pulmonologists and the immunologists. Within the quantitative model, components identified from the diagnostic model were assigned points based on the relative importance of the components in making a diagnosis. Under the point model, an infant would be considered to be a confirmed case if the points reached a certain threshold, with the thresholds being developed independently for each disorder. Each expert panel developed and applied a point model to one disorder within that panel’s expertise, applying the model to the clinical cases contributed by the members of the group. After deliberation at the in-person meeting, each group independently concluded that a point model was not feasible due to the number of factors that could influence a diagnosis. For example, laboratory results could be affected by external factors and one-point system would not be able to incorporate the external factors. The resulting categorization of a point score would either be too broad, resulting in a large number of diagnoses, or too narrow, with only a few infants receiving a diagnosis under the public health model. Agreement on the point model was reached in each of the groups independently, and the decisions were shared between groups, with the ultimate conclusion that a quantitative model was not feasible.

The metabolic and hematology panel started with a tiered model. Both groups considered cases in which there were no discrepancies about the certainty of a diagnosis of an infant with the disorder following an abnormal newborn screen. The experts determined which components of the diagnostic models were required to be known in order to conclude that a diagnosis was indisputable. This model allows for the absence or presence of different laboratory test outcomes in combination with each other in order for the diagnosis to be confirmed and allows for variation on a diagnostic evaluation based on clinical practices. For example, local clinical practices may not provide clinicians the ability to complete molecular testing on the infant, but other diagnostic components may be assembled that result in a definitive diagnosis. In the absence of best practices for standards of care or diagnosis this model provides flexibility to evaluate the certainty of the diagnosis with the available diagnostic test results and clinical evaluation. Various cases were evaluated and experts determined tiered levels of certainty that reflected the level of clinical and laboratory evidence that were available for the public health laboratory, leading to the development of a specific tiered model, the Certainty model, described below. Due to scheduling constraints the endocrine expert panel initiated their work after the other groups had made decisions. Therefore, the endocrine expert panel was provided the final model example that was developed by the metabolic group.

#### The Final Model

Following subsequent discussion and iterative deliberation, the experts concluded that a Certainty Model, a variation on the tiered model, would be most appropriate for NBS conditions. Each group discussed the merits of each of the models, and due to the limitations of the quantitative model and the relative flexibility of the tiered model, all groups determined that a specific tiered model, the certainty model, would be the best suited for all NBS disorders. Four categories of case certainty were described: Definite, Probable, Possible, and Unlikely. An Incomplete category was also established to encompass newborns with an abnormal screen where either no diagnostic work-up or additional testing was completed or insufficient data were available. A case that fell into the Incomplete category would alert the state NBS Program that short-term follow-up may not have been sufficient, prompting the program to initiate steps to ensure that the proper diagnostic work-up is completed.

Criteria were outlined to determine what would constitute a Definite, Probable, Possible, and Unlikely case for each individual NBS condition (Table 1). The participants worked under a consensus decision-making model, incorporating clinical judgment, experience, and data available to determine the certainty of disease.

A template was developed for each of the categorical determinations of diagnosis for each condition. Criteria were debated and refined through an iterative process to ensure that the definitions were comprehensive. The experts agreed that while clinical diagnosis and intervention should be initiated as early as possible, the final NBS public health surveillance case definition data collection should be completed by the time an infant is one year of age, and NBS programs could evaluate the data on an annual basis at a minimum, or more frequently as determined by the program.

### 3.2. Feedback on Case Definitions

#### 3.2.1. HRSA Regional Genetics Collaboratives

In the spring of 2012, the content of the preliminary NBS case definitions in the Certainty Model were reviewed by the membership of the HRSA-funded Regional Collaboratives (RCs) [31]. The RCs provide a regional infrastructure of public health genomics expertise to expand, strengthen, and evaluate access to a system of genetic services to improve health outcomes. The RCs were charged with reviewing the case definitions and providing feedback based upon actual cases from their respective regions using the following assumptions:

- Cases were limited to those reported to the NBS program:
  ○ Positive screens: cases detected by NBS (with a positive screen).
  ○ False negatives: clinically diagnosed cases missed by the NBS that should have been identified through NBS (not late onset of disease).
- Categorization was based on data reported in patient medical records compiled from clinicians, sub-specialists, and hospitals. NBS programs would query clinical specialists to retrospectively review the results and use the proposed categories to determine each patient’s final diagnosis. Individual case data that would increase case definition certainty would be further sought by the NBS program to establish the final diagnostic category documented for public health surveillance.

The feedback was incorporated into the case definitions by the expert workgroups prior to the July meeting of state NBS representatives.

#### 3.2.2. State NBS Program Representatives

In July 2012, a representative from each US-based NBS (follow-up and laboratory) program was invited to attend an in-person meeting in Washington, DC to assess feasibility of and attitudes towards implementing the Certainty Model case definitions at the state level. [State representatives are listed with institutional affiliations in the Supplementary Materials]. Representatives from 35 state NBS programs attended the meeting.

In preparation for this meeting and to help facilitate the upcoming discussion, participants were asked to apply the Certainty Model definitions to actual case data from their NBS programs and to only consider cases arising from NBS or those that were false negative cases that should have been identified through NBS. Attendees were not evaluating the proposed case definitions on specific clinical values or diagnostic tests, but rather on the relative ease or difficulty with which these values could be obtained by the state program from the specialty clinics. At the meeting the acceptability and feasibility of the case definitions were discussed and summarized for distribution back to the expert workgroups to further refine the Certainty Model for each condition.

### 3.3. Expert Workgroup, State and Regional Values and Preferences

Prior to implementation and pilot-testing the case definitions, the expert workgroups finalized condition specific NBS case definitions according to a Certainty Model. Stakeholder values and preferences were weighed to inform final decision making prior to implementing and piloting case definitions. Potential benefits and harms of implementation were identified. A potential benefit for clinicians was improved communication with state programs, while identified harms were a fear of less clinical autonomy and more invasive testing on newborns, such as skin biopsy. Public health NBS programs viewed benefits of standardized definitions, the ability to conduct program quality improvement and assurance, and ability to combine data across regions and nationally to conduct epidemiological analyses. Harms included the potential for extra work, although most states have an established communication network among specialty clinical programs and public health programs. Feedback from states and regional networks was taken into consideration. Implementation of case definitions, described below, incorporated suggestions on state toolkits and outreach.

### 3.4. Implementation and Piloting of Case Definitions

#### 3.4.1. Creation of a National NBS Repository

The Newborn Screening Technical assistance and Evaluation Program (NewSTEPs) is a HRSA-funded initiative with the goal of facilitating NBS related technical assistance and data collection throughout the United States [11]. This project is a continuation of efforts that were previously undertaken by the National Newborn Screening and Genetics Resource Center [32]. The case definitions developed by the expert workgroups, under the leadership of HRSA, are now integrated into the NewSTEPs data repository [11]. State NBS programs must have a Memorandum of Understanding with the Association of Public Health Laboratories (APHL) before individual case data can be entered to ensure that data privacy and data security requirements are maintained. Final case definitions are publically available at https://www.newsteps.org/quality-practice-resources/case-definitions and will be updated and maintained on this website. Modifications to the case definitions will be announced through standard avenues in the *Morbidity and Mortality Weekly Report*, NBS listservs, communication with the NBS state public health programs, and via professional meetings and publications.

#### 3.4.2. Pilot Study of Case Definitions in State NBS Programs

Beginning in late 2012 and through 2013, nine volunteer state programs piloted and validated metabolic, endocrine, and cystic fibrosis case definitions using worksheets and Research Electronic Data Capture (REDCap) (State NBS Representatives that participated in the pilot program are listed with institutional affiliations in the Supplementary Materials). This pilot study was approved by local institutional review boards, depending on local requirements. States entered case definition information for each condition from cases identified in the two previous years, with a maximum of 10 cases per condition per state. A summary of key issues with implementation of the case definitions and themes identified by state NBS programs is presented in Table 2.

#### 3.4.3. Development of Case Definitions for new disorders added to the Recommended Uniform Screening Panel

Case definitions for each new disorder added to the RUSP will be developed using a similar process to include meetings of clinical experts, feedback from state newborn screening programs, and integration into the NewSTEPs data repository. To date, this process has been completed for critical congenital heart (CCHD), Pompe disease, Mucopolysaccharidosis Type-I (MPS I), and X-linked Adrenoleukodystrophy (XALD). Case definitions for each future condition will be developed following the formal approval by the Secretary of Health and Human Services.

### 3.5. Dissemination Plan for NBS Case Definitions

Dissemination of the case definitions for NBS is being completed through a partnership of many organizations and venues. The overarching strategy includes presentations and educational events at national and regional meetings, including the Secretary’s Advisory Committee on Heritable Disorders in Newborns and Children meeting, APHL Newborn Screening and Genetic Testing Symposium, Association of Maternal and Child Health Programs Annual Conference, ACMG Annual Meeting, and Regional Genetic and Newborn Screening Service Collaborative meetings. Broader awareness of the NBS case definitions has been achieved through the ongoing review and piloting cycles described above, and through presentation to broad audiences that include public health programs and clinical specialists. State NBS programs are encouraged to utilize the case definitions and to enter case data into the NewSTEPs repository. NewSTEPs also led webinar tutorials for state NBS programs on how to implement case definitions and how to access the case definition Supplementary Materials.

#### State Implementation Toolkit

To facilitate case data entry, NewSTEPs developed a case definition Supplementary Materials webpage that provides resources about the public health surveillance case definitions for NBS (https://www.newsteps.org/case-definitions). These resources include case definition worksheets, classification tables, and a case definition toolkit. The case definition worksheets provide a systematic approach to the questions required to compete the case definitions. The worksheets follow a sequential pattern, mirroring the accompanying disease classification tables. The classification tables help to identify the level of certainty of a case as Definite, Probable, Possible, Unlikely, or Incomplete based on different criteria established by the expert workgroups.

The case definition toolkit was developed at the request of those state NBS programs that participated in pilot testing the case definitions. Some state NBS programs may not have an established or strong relationship with the medical providers who care for the children diagnosed with a NBS condition. The toolkit contains a variety of documents that will aid the state NBS programs in gathering the necessary information to complete the worksheets. They are described below:

- Purpose of Case Definitions: A document describing the need for case definitions, the limitations of current practices, the need for short- and long-term follow-up in public health surveillance, the information NewSTEPs is collecting, how it will be utilized, and the benefit to state NBS programs.
- A Sample Letter of Introduction: For medical providers from the state NBS program to obtain necessary diagnostic information.
- Lessons Learned: A document providing tips and advice from the pilot state NBS programs to other state NBS programs to guide them as they start to implement the case definitions.

Each NBS case will be entered through a Java-Script enabled secure SQL server database in the NewSTEPs repository. Partnering with the Laboratory Information Management Systems (LIMS) vendors, NewSTEPs has developed and continues to refine a tool for NBS programs to extract infant level data (during the first year of life) from the LIMS systems and upload data into the NewSTEPs data repository. Algorithms calculate the certainty of the diagnosis based on the tables developed by the expert clinicians. This provides consistent implementation of the case definition tables across all NBS programs.

### 3.6. Evaluation of Case Definitions and Surveillance Process

The performance of the case definitions will be measured by (1) their utility for NBS programs and (2) the ability of the definitions to support public health surveillance across states. Utility will include the simplicity, flexibility, acceptability, and timeliness of the case definitions [33]. Currently, many programs do not collect detailed diagnostic information to appropriately categorize each case according the public health surveillance definitions. Doing so will involve considerable effort, time, and resources. Sufficient data collection in order to calculate the case definition certainty is necessary for the evaluation of the performance of the algorithms.

Evaluation of case definitions will need to proceed in stages, by condition, starting with an evaluation of completeness and timeliness. Following two years of data entry (2013 and 2014 birth years) and on an ongoing three-year recurring cycle, aggregate data will be shared with the clinical expert teams to assess if the case definitions have performed as anticipated, utilizing measures of data quality, representativeness, and stability. It is crucial for data quality that the definitions are acceptable to the community entering the data, and that the system is representative of the United States. To assess acceptability of the case definitions, the NBS community will be surveyed and focus groups will be completed. The representativeness of the case definitions will be assessed through comparison of cases reported to NewSTEPs using the case definitions and expected frequencies of cases, and through comparison to frequencies reported to clinical registries. Following each three-year cycle, the case definitions will be reviewed and modifications to the case definitions will be made, as needed. Case definitions for new disorders will be developed as they are added to the RUSP. Case definition tables are available on the NewSTEPs website (https://www.newsteps.org/case-definitions).

Comparisons between the NBS program’s interpretation of the definition and the automated calculation of the certainty will be performed to assess NBS program understanding and implementation of the case definitions. Aggregate data will be available on the NewSTEPs website. Individuals wishing to query the data system and the cases identified across states can do so through a formal data query process as outlined on the NewSTEPs website. The evaluation of completeness through NewSTEPs will identify patterns of missing data that may point to particularly challenging data to collect and report.

## 4. Discussion

The case definitions presented here were developed through a structured process over the course of two years that involved specialists in metabolic disorders, endocrinology, pulmonology, hematology, and state NBS program personnel. These definitions are specifically designed for state NBS programs to consistently and uniformly identify, characterize, and monitor the disorders included on the RUSP. NBS programs will categorize infants who have received a clinical diagnosis following an out-of-range newborn screen using the available clinical diagnostic information applied to these case definitions. The novel case definitions standardize a process that already exists between state NBS programs and clinical specialists. Therefore, the need for additional resources is minimized. The public health surveillance case definitions are not intended to alter or influence diagnostic decisions or clinical care, which rely on a more complex set of clinical variables generated by each individual clinical setting and presentation and are interpreted by a clinical practitioner. The public health surveillance definitions are agnostic to varying clinical opinion and practices; infants will be categorized in the same manner regardless of regional and period effects that may result in different clinical care and diagnostic decisions.

The development of these case definitions has several limitations. First, a systematic literature review of case definitions in other fields was not completed, therefore other viable models for newborn screening case definitions may not have been considered. Second, the case definitions that were developed have not yet been validated and will be validated through the evaluation process described above. Finally, the voluntary nature of data entry into the NewSTEPs Repository may impede our ability to validate case definitions, particularly for very rare disorders.

## 5. Conclusions

This report presents novel case definitions for use by public health NBS programs. These definitions were developed through expert opinion and refined by other stakeholder feedback. These case definitions facilitate consistent data collection, comparison, and analysis of cases identified through newborn screening across states, regions, and at a national level. As case data are evaluated by NewSTEPS, these definitions will be updated.

## Figures and Tables

**Table 1:** Criteria for certainty categories for public health surveillance case definitions for newborn screening.

Certainty Category	General Criteria
Definite case	All providers reviewing the result(s) would be confident in the diagnosis.
Probable Case	Evidence to the NBS program suggests the presence of the condition, but one or more confirmatory diagnostic elements are unavailable.
Possible Case	Very limited confirmatory data available to the state NBS program, but the available data do not rule out the diagnosis.
Unlikely	Evidence to the NBS program suggests a carrier status or absence of a condition.

NBS = newborn screening.

**Table 2:** Summary of themes identified by state newborn screening programs participating in pilot study of case definitions (October 2012–September 2013).

Theme Identified by State NBS Program	Solution Implemented	Example of Implemented Change *(25)*
Challenges in contacting clinicians/getting buy-in to provide requested information	Letter template developed for use by state NBS programs for communicating with clinicians	Toolkit Content
Data privacy and security for national collection of case definition data (in a system such as the NewSTEPs data repository)	Transparent policies on data use and storage policies for national data collection; publically sharing Institutional Review Board decision and Office of Human Research Protection discussions regarding data use for national repository	Toolkit Content
Clinical information requested that was not required for collection at state level; States unsure of authority to collect	Discussion of necessity of state public health programs to collect basic information in categories to close cases	Toolkit Content
Challenges in entering clinical laboratory results: requires reference ranges and units	Laboratory results were made categorical	Case Definition Worksheets
Inconsistencies in mutation nomenclature; Mutations presented in accordance with the Human Genome Variation Society standards while others presented in traditional format	Case definitions modified to describe functional status (e.g., mutation known to be disease causing, variant of unknown significance)	Case Definition Worksheets
Logic and structure of data collection forms	Modification of forms to facilitate data collection	Case Definition Worksheets

NBS = newborn screening; NewSTEPs = Newborn Screening Technical assistance and Evaluation Program.

## Supplementary Materials

The following are available online at http://www.mdpi.com/2409-515X/4/2/16/s1.
