# Supplementary material for: Case Definitions for Conditions Identified by Newborn Screening Public Health Surveillance
Source: Int J Neonatal Screen. 2018 May 9;4(2):16. doi: 10.3390/ijns4020016 (PMC5978752; doi:10.3390/ijns4020016)
Supplement: Supplementary file 1 [file IJNS-04-00016-s001.pdf]

Article

Case Definitions for Conditions Identified by Newborn Screening Public Health Surveillance

Marci K. Sontag [1,2,*], Deboshree Sarkar [3], Anne M. Comeau [4], Kathryn Hassell [5], Lorenzo D. Botto [6], Richard Parad [7], Susan R. Rose [8], Kupper A. Wintergerst [9], Kim Smith-Whitley [10], Sikha Singh [2,11], Careema Yusuf [2,11], Jelili Ojodu [2,11], Sara Copeland [12] and Cynthia F. Hinton [13,†]

1 Department of Epidemiology, Colorado School of Public Health, University of Colorado Anschutz Medical Campus, Aurora, CO 80045, USA
2 NewSTEPs, Newborn Screening Technical assistance and Evaluation Program, A Program of the Association of Public Health Laboratories, Silver Spring, MD 20910, USA; sikha.singh@aphl.org (S.S.); careema.yusuf@aphl.org (C.Y.); jelili.ojodu@aphl.org (J.O.)
3 Health Resources and Services Administration, Maternal Child Health Bureau, Division of Services for Children with Special Health Needs, Genetic Services Branch, Rockville, MD 20852, USA; DSarkar@hrsa.gov
4 New England Newborn Screening Program, University of Massachusetts Medical School, Worcester, MA 01605, USA; Anne.Comeau@umassmed.edu
5 Division of Hematology, University of Colorado School of Medicine, University of Colorado Denver Anschutz Medical Campus, Aurora, CO 80045, USA; kathryn.hassell@ucdenver.edu
6 Department of Pediatrics, University of Utah School of Medicine, Salt Lake City, UT 84132, USA; Lorenzo.Botto@hsc.utah.edu
7 Department of Pediatrics, Harvard Medical School Department of Pediatric and Newborn Medicine, Brigham and Women's Hospital, Boston, MA 02115, USA; rparad@bwh.harvard.edu
8 Endocrinology and Pediatrics, Cincinnati Children's Hospital Medical Center, University of Cincinnati College of Medicine, Cincinnati, OH 45229, USA; mslrose4@gmail.com
9 Division of Endocrinology, Department of Pediatrics, School of Medicine, University of Louisville, Louisville, KY 40202, USA; kupper.wintergerst@louisville.edu
10 Division of Hematology, Children's Hospital of Philadelphia, Department of Pediatrics, Perelman School of Medicine, Philadelphia, PA 19104, USA; whitleyk@email.chop.edu
11 Association of Public Health Laboratories, Silver Spring, MD 20910, USA
12 Palo Alto Medical Foundation, Daly City, CA 94015, USA; saracopeland72@gmail.com
13 National Center on Birth Defects and Developmental Disabilities, Centers for Disease Control and Prevention, Atlanta, GA 30341, USA; ceh9@cdc.gov
* Correspondence: marci.sontag@ucdenver.edu
† On behalf of the expert advisor working group.

Supplementary Materials

Case Definition Oversight Committee

- Sara Copeland, M.D., Maternal and Child Health Bureau, Health Resources and Services Administration, Rockville, Maryland (previous). Palo Alto Medical Foundation, Daly City, California (current).
- Cynthia Hinton, Ph.D., M.S., M.P.H., National Center on Birth Defects and Developmental Disabilities, Centers for Disease Control and Prevention, Atlanta, Georgia.
- Richard Olney, M.D., M.P.H., Division of Birth Defects and Developmental Disabilities, Centers for Disease Control and Prevention, Atlanta, Georgia.
- Melissa Parisi, M.D., Ph.D., Intellectual and Developmental Disabilities (IDD) Branch, *Eunice Kennedy Shriver* National Institute of Child Health and Human Development, National Institutes of Health, Bethesda, Maryland.

- Deboshree Sarkar, M.P.H., Maternal and Child Health Bureau, Health Resources and Services Administration, Rockville, Maryland.
- Tiina Urv, Ph.D., National Institute for Child Health and Human Development, Intellectual and Developmental Disabilities (IDD) Branch, Bethesda, Maryland.

NBS Case Definitions Expert Workgroup Members

- Jose Abdenur, M.D., Division of Metabolic Disorders at Children's Hospital of Orange County, Orange, California.
- Swapna Abhyankar, M.D., Lister Hill National Center for Biomedical Communications, U.S. National Library of Medicine, Bethesda, Maryland (previous); Regenstreif Institute, Indianapolis, Indiana (current).
- Frank Accurso, M.D., Departments of Pediatrics and Physiology and Biophysics, University of Colorado School of Medicine, Department Epidemiology, Colorado School of Public Health, University of Colorado Denver Anschutz Medial Campus, Aurora, Colorado.
- Susan Berry, M.D., Pediatric Genetic and Metabolics, University of Minnesota, Minneapolis, Minnesota.
- Vincent Bonagura, M.D., Department of Allergy/Immunology, Long Island Jewish Medical Center, Great Neck, New York
- Francisco Bonilla, M.D., Ph.D., Department of Pediatrics, Harvard Medical School, Boston Children's Hospital Division of Immunology, Boston Massachusetts.
- Drucy Borowitz, M.D., Department of Pediatrics, Women and Children's Hospital of Buffalo Division of Pulmonology, Buffalo, New York.
- Lorenzo Botto, M.D., Division of Medical Genetics, Department of Pediatrics, University of Utah School of Medicine, Salt Lake City, Utah.
- Amy Brower, Ph.D., American College of Medical Genetics and Genomics, Bethesda, Maryland.
- Rebecca Buckley, M.D., Department of Pediatrics, Department of Immunology, Duke University School of Medicine, Durham, North Carolina.
- Anne Marie Comeau, Ph.D. New England Newborn Screening Program, University of Massachusetts Medical School, Worcester, Massachusetts.
- Carla Cuthbert, Ph.D., F.A.C.M.G., F.C.C.M.G., Newborn Screening and Molecular Biology Branch, Division of Laboratory Sciences, Centers for Disease Control and Prevention, Atlanta, Georgia.
- Hank Dorkin, M.D., Division of Respiratory Diseases, Harvard Medical School, Boston Children's Hospital, Boston, Massachusetts.
- Jim Eckman, M.D., Professor Emeritus, Emory University School of Medicine, Atlanta, Georgia.
- Phil Farrell, M.D., Departments of Pediatrics and Population Health Sciences, University of Wisconsin School of Medicine and Public Health, Madison, Wisconsin.
- Rebecca Goodwin, J.D., Lister Hill National Center for Biomedical Communications, U.S. National Library of Medicine, Bethesda, Maryland.
- Dan Hale, M.D., Division of Endocrinology & Diabetes, University of Texas Health Sciences Center, San Antonio, Texas.
- Cary Harding, M.D., School of Medicine, Oregon Health & Science University, Portland, Oregon.
- Kathy Hassell, M.D., Division of Hematology, University of Colorado School of Medicine, University of Colorado Denver Anschutz Medical Campus, Aurora, Colorado.
- Carolyn Hoppe, M.D., University of California San Francisco Benioff Children's Hospital Oakland, Oakland, California.
- Michelle Howenstine, M.D., Pediatric Pulmonary, Allergy and Sleep Medicine, Riley Hospital for Children at IU Health, Indianapolis, Indiana.
- Stephen Kahler, M.D., College of Medicine, Department of Pediatrics, University of Arkansas for Medical Sciences, Little Rock, Arkansas.

- Celia Kaye, M.D., Ph.D., Department of Pediatrics, School of Medicine, University of Colorado Denver, Anschutz Medical Campus, Aurora, Colorado, and Department of Pediatrics at the University of Texas Health Science Center, San Antonio, Texas.
- David Kronn, M.D., Inherited Metabolic Disease Center, Westchester Medical Center, Departments of Pathology and Pediatrics, New York Medical College, Valhalla, New York.
- Ferdane Kutlar, M.D., Hematology/Oncology Section, THJ Huisman Hemoglobinopathy / Genetic Testing Laboratory, Medical College of Georgia at Georgia Regents University, Augusta, Georgia.
- Stephen LaFranchi, M.D., Department of Pediatrics, Division of Pediatric Endocrinology, Doernbecher Children's Hospital /Oregon Health & Science University, Portland, Oregon.
- Nancy Leslie, M.D., Division of Human Genetics, Department of Pediatrics, Cincinnati Children's Hospital Medical Center, Cincinnati, Ohio.
- Sean McGhee, M.D., Pediatric Allergy and Immunology, Stanford School of Medicine, Palo Alto, California.
- Maddy Martin, M.D., Department of Pediatrics, University of Massachusetts Memorial Medical Center, Worcester, Massachusetts.
- Marvin Mitchell, M.D., University of Massachusetts Medical School, New England Newborn Screening Program, Worcester, Massachusetts.
- Richard Olney, M.D., M.P.H., Division of Birth Defects and Developmental Disabilities, Centers for Disease Control and Prevention, Atlanta, Georgia.
- Richard Parad, M.D., M.P.H., Department of Pediatric Newborn Medicine, Harvard Medical School, Brigham and Women's Hospital, Boston, Massachusetts.
- Melissa Parisi, M.D., Ph.D., Intellectual and Developmental Disabilities (IDD) Branch, *Eunice Kennedy Shriver* National Institute of Child Health and Human Development, National Institutes of Health, Bethesda, Maryland.
- Chanika Phornphutkul, M.D., Department of Pediatrics Warren Alpert Medical School of Brown University, Hasbro Children's Hospital, Providence, Rhode Island.
- Jennifer Puck, M.D., Department of Pediatrics, University of California School of Medicine, San Francisco, California.
- George Retsch-Bogart, M.D., Division of Pediatric Pulmonology, University of North Carolina at Chapel Hill, Chapel Hill, North Carolina.
- Michael Rock, M.D., Department of Pediatrics, University of Wisconsin School of Medicine and Public Health, Madison, Wisconsin.
- Susan R. Rose, M.D., Endocrinology and Pediatrics, Cincinnati Children's Hospital Medical Center, University of Cincinnati College of Medicine, Cincinnati, Ohio.
- John M. Routes, M.D., Section of Allergy and Clinical Immunology, Departments of Pediatrics, Medicine, Microbiology and Molecular Genetics, Children's Hospital of Wisconsin Medical College of Wisconsin, Milwaukee, Wisconsin.
- Kim Smith-Whitley, M.D., Division of Hematology, Children's Hospital of Philadelphia, Department of Pediatrics, Perelman School of Medicine, Philadelphia, Pennsylvania.
- Phyllis W. Speiser, M.D., Division of Pediatric Endocrinology, Cohen Children's Medical Center of New York, Hofstra North Shore-LIJ School of Medicine, Lake Success, New York.
- Brad Therrell, Ph.D. National Newborn Screening and Global Resource Center, Austin, Texas.
- Janet Thomas, M.D., Division of Clinical Genetics & Metabolism, Department of Pediatrics, School of Medicine, University of Colorado Denver, Anschutz Medical Campus, Aurora, Colorado.
- Tiina Urv, Ph.D., National Institute for Child Health and Human Development, Intellectual and Developmental Disabilities (IDD) Branch, Bethesda Maryland.
- Laurie Varlotta, M.D., Section of Pediatric Pulmonology, St. Christopher's Hospital for Children, Philadelphia, Pennsylvania.
- Elliott Vichinsky, M.D., Department of Pediatrics, University of California San Francisco School of Medicine, UCSF Benioff Children's Hospital Oakland, Oakland, California.

- Ellen Werner, Ph.D., M.A., Division of Blood Diseases and Blood Resources, National Heart, Lung, and Blood Institute, National Institutes of Health Bethesda, Maryland.
- Kupper Wintergerst, M.D., Division of Endocrinology, Department of Pediatrics, University of Louisville, School of Medicine, Louisville, Kentucky.
- Roberto Zori, M.D., Division of Genetics and Metabolism, University of Florida College of Medicine, Gainesville, Florida.

Disclosure of Competing Interests: The developers of these guidelines wish to disclose that they have no financial interests or other competing interests with commercial products or suppliers related to the development of these newborn screening surveillance case definitions.

Regional Genetic and Newborn Screening Services Collaboratives

- Region 1: New England Genetics Collaborative
  Connecticut, Maine, New Hampshire, Rhode Island, and Vermont
- Region 2: New York Mid-Atlantic Collaborative
  District of Columbia, Delaware, Maryland, New Jersey, New York, Pennsylvania, Virginia, and West Virginia
- Region 3: Southeast Regional Collaborative
  Alabama, Florida, Georgia, Louisiana, Mississippi, North Carolina, Puerto Rico, South Carolina, Tennessee, and U.S. Virgin Islands
- Region 4: Midwest Genetics Collaborative
  Illinois, Indiana, Kentucky, Michigan, Minnesota, Ohio, and Wisconsin
- Region 5: Heartland Genetics and Newborn Screening Collaborative Arkansas, Iowa, Kansas, Missouri, Nebraska, North Dakota, Oklahoma, and South Dakota
- Region 6: Mountain States Genetics Regional Collaborative
  Arizona, Colorado, Montana, New Mexico, Nevada, Texas, Utah, and Wyoming
- Region 7: Western States Genetic Services Collaborative
  Alaska, California, Guam, Hawaii, Idaho, Oregon, and Washington

Participants in the 2012 Stakeholder Meeting: Swapna Abhyankar, M.D., Lister Hill National Center for Biomedical Communications, U.S. National Library of Medicine, Bethesda, Maryland (previous); Regenstreif Institute, Indianapolis, Indiana (current).

- Cindy Ashley, R.N., B.S.N.c, Alabama Department of Public Health, Montgomery, Alabama.
- Becky Bailey, R.N., B.N.S.c, North Dakota Department of Health, Bismarck, North Dakota.
- Louis Bartoshesky, M.D., M.P.H., Christiana Care, Newark, Delaware.
- Linda Beischel, C.L.S.p (M.B.), Montana Department of Public Health and Human Services, Helena, Montana.
- Stan Berberich, Ph.D., State Hygienic Laboratory at the University of Iowa, Ankeny, Iowa.
- Natasha Bonhomme, Baby's First Test, Genetic Alliance, Washington, DC.
- Bob Bowman, M.S., Indiana State Department of Health, Indianapolis, Indiana.
- Amy Brower, Ph.D., American College of Medical Genetics and Genomics, Bethesda, Maryland.
- Michele Caggana, Sc.D., FACMG, New York State Department of Health, Wadsworth Center, Albany, New York.
- Colleen Clarke, C.L.S., Louisiana Department of Health & Hospitals, New Orleans, Louisiana.
- Anne Marie Comeau, Ph.D. New England Newborn Screening Program, University of Massachusetts Medical School, Worcester, Massachusetts.
- Sara Copeland, M.D., Maternal and Child Health Bureau, Health Resources and Services Administration, Rockville, Maryland (previous). Palo Alto Medical Foundation, Daly City, California (current).
- William Cramer, M.Ed., Pennsylvania Department of Health, Harrisburg, Pennsylvania.
- Hank Dorkin, M.D., Division of Respiratory Diseases, Harvard Medical School, Boston Children's Hospital, Boston, Massachusetts
- Roger Eaton, Ph.D., New England Newborn Screening Program, University of Massachusetts Medical School, Jamaica Plain, Massachusetts.

- Lisa Feuchtbaum, Dr.P.H., M.P.H., California Department of Public Health, Richmond, California.
- Bryant Fortner, M.D., University Primary Care, Columbia, South Carolina.
- Lucy Fossen, R.N., South Dakota Department of Health, Pierre, South Dakota.
- Debra Freedenberg, M.D., Ph.D., Texas Department of State Health Services, Austin, Texas.
- Jane Getchell, Dr.P.H., Association of Public Health Laboratories, Silver Spring, Maryland.
- Michael Glass, M.S., Washington Department of Health, Shoreline, Washington.
- Aaron Goldenberg, Ph.D., M.P.H., Case Western Reserve University, Cleveland, Ohio.
- Arthur Hagar, Ph.D., Georgia Public Health Laboratory, Atlanta, Georgia.
- Deboshree Sarkar, M.P.H., Maternal and Child Health Bureau, Health Resources and Services Administration, Rockville, Maryland.
- Kathy Hassell, M.D., Division of Hematology, University of Colorado School of Medicine, University of Colorado Denver Anschutz Medical Campus, Aurora, Colorado.
- Cynthia Hinton, Ph.D., M.S., M.P.H., National Center on Birth Defects and Developmental Disabilities, Centers for Disease Control and Prevention, Atlanta, Georgia.
- Amy Hoffman, M.P.H., American College of Medical Genetics and Genomics, Bethesda, Maryland.
- Philis Hoggatt, R.N., Mississippi Department of Health, Jackson, Mississippi.
- Patrick Hopkins, Missouri State Public Health Laboratory, Jefferson City, Missouri.
- Cynthia Ingham, R.N., B.S.N., Vermont Department of Health, Burlington, Vermont.
- Ward Jacox, Arizona State Public Health Laboratory, Phoenix, Arizona.
- Carol Johnson, Iowa Newborn Screening Program, University of Iowa Children's Hospital, Iowa City, Iowa.
- Yvonne Kellar-Guenther, Ph.D., Colorado School of Public Health, University of Colorado Denver, Anschutz Medical Campus, Aurora, Colorado.
- Jamey Kendall, R.N., B.S.N., Kansas Department of Health and Environment, Topeka, KS.
- Janice Kong, M.T., Hawaii Department of Health, Honolulu, Hawaii.
- Michelle Lewis, M.D., JD, Berman Institute of Bioethics, Johns Hopkins University, Washington, DC.
- Sharon Linard, M.S., Ohio Department of Health, Reynoldsburg, Ohio.
- Jennifer Macdonald, R.N., B.S.N, M.P.H., Virginia Department of Health, Richmond, Virginia.
- Mark McCann, Minnesota Department of Health, St. Paul, Minnesota.
- Jelili Ojodu, M.P.H., Association of Public Health Laboratories, Silver Spring, Maryland.
- Susan Oliver, M.S.N., R.N., Connecticut Department of Public Health, Hartford, Connecticut.
- Richard Parad, M.D., M.P.H., Harvard Medical School, Brigham and Women's Hospital, Boston Children's Hospital, Boston, Massachusetts.
- Melissa Parisi, M.D., Ph.D., Intellectual and Developmental Disabilities (IDD) Branch, *Eunice Kennedy Shriver* National Institute of Child Health and Human Development, National Institutes of Health, Bethesda, Maryland.
- Julie Raburn-Miller, M.S.W., L.C.S.W., Department of Health and Senior Services, Bureau of Genetics and Healthy Childhood, Jefferson City, Missouri.
- Deborah Rodriguez, RN, M.P.H., CPH, New York State Department of Health, Wadsworth Center, Albany, New York.
- Inderneel Sahai, M.D., New England Newborn Screening Program, Massachusetts General Hospital, Boston, Massachusetts.
- Scott Shone, Ph.D., New Jersey Department of Health, Trenton, New Jersey (previous); RTI International, Research Triangle Park, North Carolina (current).
- Marci Sontag, Ph.D., Colorado School of Public Health, University of Colorado Denver, Anschutz Medical Campus, Aurora, Colorado.
- Susan Tanksley, Ph.D., Texas Department of State Health Services, Austin, Texas.
- Laura Taylor, Colorado Department of Public Health & Environment, Denver, Colorado.
- Lois Taylor, R.N., B.S.N., C.P.M., Florida Department of Health. Tallahassee, Florida.
- Patricia Terry, M.S.M., L.S.W., Mississippi State Department of Health, Jackson, Mississippi.

- Tiina Urv, Ph.D., Eunice Kennedy Shriver National Institute of Child Health and Human Development, National Institutes of Health
- Sheila Weiss, M.S., Washington State Department of Health, Shoreline, Washington.
- Kupper Wintergerst, M.D., Department of Pediatrics, Division of Endocrinology, University of Louisville, School of Medicine, Louisville, Kentucky.
- Alan Zuckerman, M.D., Lister Hill National Center for Biomedical Communications, U.S. National Library of Medicine, Bethesda, Maryland
- Cindy Ashley, B.S.N., R.N.c, Alabama Newborn Screening Program, Montgomery, Alabama.

Delaware

- Louis Bartoshesky, M.D., M.P.H., Christiana Care, Newark, Delaware.

Hawaii

- Janice Kong, M.T., Hawaii Department of Health, Honolulu, Hawaii.

Iowa

- Carol Johnson, Iowa Newborn Screening Program, University of Iowa Children's Hospital, Iowa City, Iowa.

Kansas

- Jamey Kendall, R.N., B.S.N., Kansas Department of Health and Environment, Topeka, KS.
- Louisiana
- Cheryl Harris, M.P.H., Louisiana Department of Health, New Orleans, Louisiana.
- Colleen Clarke, C.L.S., Louisiana Department of Health & Hospitals, New Orleans, Louisiana.

Maryland

- Debbie Badawi, M.D., Maryland Department of Health and Mental Hygiene, Baltimore, Maryland.
- Johnna L. Watson, Maryland Department of Health and Mental Hygiene, Baltimore, Maryland.
- Donna X. Harris, Maryland Department of Health and Mental Hygiene, Baltimore, Maryland.

Missouri

- Julie Raburn-Miller, M.S.W., L.C.S.W., Missouri State Public Health Laboratory, Jefferson City, Missouri.
- Jami Kiesling, R.N., B.S.N., Department of Health and Senior Services, Bureau of Genetics and Healthy Childhood, Jefferson City, Missouri.
- Patrick Hopkins, Missouri State Public Health Laboratory, Jefferson City, Missouri.

Nebraska

- Julie Luedtke, Nebraska Department of Health & Human Services, Lincoln, Nebraska.
- Krystal Baumert, Nebraska Department of Health & Human Services, Lincoln, Nebraska
- Karen Eavans, M.D., Nebraska Department of Health & Human Services, Lincoln, Nebraska.

Utah

- Kim Hart, Utah Department of Health State Laboratory, Salt Lake City, Utah.
